# Molecular characterization of carbapenem-resistance in Gram-negative isolates obtained from clinical samples at Jimma Medical Center, Ethiopia

**DOI:** 10.3389/fmicb.2024.1336387

**Published:** 2024-01-24

**Authors:** Mulatu Gashaw, Esayas Kebede Gudina, Solomon Ali, Liegl Gabriele, Thomas Seeholzer, Bikila Alemu, Guenter Froeschl, Arne Kroidl, Andreas Wieser

**Affiliations:** ^1^School of Medical Laboratory Sciences, Jimma University, Jimma, Ethiopia; ^2^CIHLMU Center for International Health, Ludwig Maximilians Universität München, Munich, Germany; ^3^Department of Internal Medicine, Jimma University, Jimma, Ethiopia; ^4^Saint Paul’s Hospital Millennium Medical College, Addis Ababa, Ethiopia; ^5^Max von Pettenkofer-Institute (Medical Microbiology), Ludwig Maximilian University of Munich, Munich, Germany; ^6^Fraunhofer Institute for Translational Medicine and Pharmacology ITMP, Immunology, Infection and Pandemic Research, Munich, Germany; ^7^Division of Infectious Disease and Tropical Medicine, University Hospital (LMU), Munich, Germany; ^8^German Center for Infection Research (DZIF), Munich, Germany

**Keywords:** carbapenem-resistant, carbapenemases, *bla*OXA, *bla*NDM, ESBL, Jimma

## Abstract

**Background:**

In resource-constrained settings, limited antibiotic options make treating carbapenem-resistant bacterial infections difficult for healthcare providers. This study aimed to assess carbapenemase expression in Gram-negative bacteria isolated from clinical samples in Jimma, Ethiopia.

**Methods:**

A cross-sectional study was conducted to assess carbapenemase expression in Gram-negative bacteria isolated from patients attending Jimma Medical Center. Totally, 846 Gram-negative bacteria were isolated and identified using matrix-assisted laser desorption ionization-time of flight mass spectrometry (MALDI-TOF MS). Phenotypic antibiotic resistance patterns were determined using the Kirby-Bauer disk diffusion method and Etest strips. Extended-spectrum β-lactamase phenotype was determined using MAST disks, and carbapenemases were characterized using multiplex polymerase chain reactions (PCR).

**Results:**

Among the isolates, 19% (157/846) showed phenotypic resistance to carbapenem antibiotics. PCR analysis revealed that at least one carbapenemase gene was detected in 69% (107/155) of these strains. The most frequently detected acquired genes were *bla*NDM in 35% (37/107), *bla*VIM in 24% (26/107), and *bla*KPC42 in 13% (14/107) of the isolates. Coexistence of two or more acquired genes was observed in 31% (33/107) of the isolates. The most common coexisting acquired genes were *bla*NDM + *bla*OXA-23, detected in 24% (8/33) of these isolates. No carbapenemase-encoding genes could be detected in 31% (48/155) of carbapenem-resistant isolates, with *P. aeruginosa* accounting for 85% (41/48) thereof.

**Conclusion:**

This study revealed high and incremental rates of carbapenem-resistant bacteria in clinical samples with various carbapenemase-encoding genes. This imposes a severe challenge to effective patient care in the context of already limited treatment options against Gram-negative bacterial infections in resource-constrained settings.

## Introduction

Gram-negative bacteria (GNB), such as *Escherichia coli*, *Klebsiella pneumoniae*, *Acinetobacter baumannii*, and *Pseudomonas aeruginosa*, are common culprits in healthcare-associated infections (Sikora and Zahra, 2020). Carbapenem resistance is increasing at alarming rates in these organisms (Beshah et al., 2023). The resistance can arise from various mechanisms, including the production of carbapenemase enzymes, decreased permeability of the bacterial cell wall, increased efflux pump activity, alterations in outer membrane porins, and target site mutations that reduce affinity to carbapenems (Aurilio et al., 2022). These mechanisms can act individually or in combination, leading to the development of multidrug-resistant strains that pose significant challenges in treating infections caused by these bacteria (Das, 2023). GNB have the ability to acquire and express a variety of carbapenemase genes (Dwomoh et al., 2022; Tenover et al., 2022; Tilahun et al., 2022). These genes can spread within or between different bacterial species through horizontal transfer of plasmids, conjugative transposons, or integrons (Hammoudi Halat and Ayoub Moubareck, 2020). As a result, carbapenem resistance in GNB is a major public-health concern worldwide. The most common carbapenemases identified in GNB include oxacillinases (OXA), *Klebsiella pneumoniae* carbapenemase (KPCs), and metallo-beta-lactamases (MBLs), including New Delhi metallo-β-lactamase (NDM) and Verona integron-encoded metallo-beta-lactamase imipenemase (VIM) (Rabaan et al., 2022). These enzymes can break down carbapenem antibiotics, and develop resistance not only to carbapenems, but also to many other beta-lactam antibiotics, such as penicillins, cephalosporins, and monobactams (Jean et al., 2022).

Infections with these pathogens are associated with high rates of mortality and morbidity since treatment options are limited to a few last-resort antibiotics that often come with many side effects (Caston et al., 2022). Furthermore, infections with carbapenem-resistant GNBs increase healthcare cost and the length of hospital stays (Van Duin, 2017). Such infections are major concerns for critically ill patients, immunocompromised individuals, and those with comorbidities (Aleidan et al., 2021; Di Carlo et al., 2021). In resource-constrained countries, including Ethiopia, the public health impact is even worse due to the lack of reserve treatment options (Alemayehu et al., 2023; Beshah et al., 2023).

Rapid and reliable detection of carbapenem-resistant GNB is critical for appropriate laboratory-guided patient management, for surveillance, and for applying effective evidence-based infection prevention and control practices (Nordmann and Poirel, 2019; Shanmugakani et al., 2020). A combination of phenotypic detection and genotypic confirmation of carbapenemase-expressing genes by polymerase chain reaction (PCR) is recommended (Rabaan et al., 2022).

However, due to lack of technical expertise, specialized equipment, and reagents, detecting and tracking the molecular epidemiology of carbapenem-resistant bacterial isolates is difficult in low-income countries (Nordmann and Poirel, 2019; Shanmugakani et al., 2020). As a result, data on the burden of infections with carbapenem-resistant bacterial species and associated outcomes is scarce in Sub-Saharan African countries, including Ethiopia (Stewardson et al., 2019). Therefore, this study aimed to determine the extent of carbapenemases among GNBs obtained from clinical samples using both phenotypic and genotypic techniques.

## Materials and methods

### Study setting, design, and time

A cross-sectional study was conducted to detect the carbapenemase genes in carbapenem-resistant GNB obtained from patients treated at Jimma Medical Center (JMC). JMC is an 800-bed teaching hospital in southwest Ethiopia with a catchment population of over 20 million. All patients from whom samples were sent for culture and antibiotic susceptibility test as part of routine clinical care were recruited prospectively for the study.

### Clinical sample collection

Clinical samples (blood, cerebrospinal fluid [CSF], wound swabs, ascitic fluid, pleural fluid, abscess, peritoneal fluid, and synovial fluid) were collected aseptically by the clinicians, nurses or laboratory professionals. Other clinical samples such as urine, stool, and sputum were collected by the patients themselves after proper instruction was provided. Samples were then transported within 1 h after collection to the JMC microbiology laboratory for analysis.

### Bacterial isolation and identification

All clinical specimens, except for blood, were inoculated on 5% Colombia Sheep Blood, Chocolate, and MacConkey agars and incubated aerobically at 35–37°C for 18–22 h. Blood samples were collected and added to BD BACTEC bottles (Becton Dickinson, Sparks, MD, USA) and then incubated for 5 days at 35–37°C in the BD BACTEC™ FX40 (Becton Dickinson, Sparks, MD, USA) automated culture machine. If growth was observed, it was sub-cultured on 5% Colombia Sheep Blood, Chocolate, and MacConkey agars in similar environmental conditions for further analysis. Subsequently, all positive pure cultures were tested for antimicrobial susceptibility. Isolates were picked off the plates and kept at −80°C in storage media containing skimmed milk, tryptone soya, glucose, glycerol, and distilled water until they were transported to Max von Pettenkofer Institute, Hospital Hygiene, and Medical Microbiology Laboratory in Munich, Germany. There, the isolates were re-identified using matrix-assisted laser desorption ionization-time of flight mass spectrometry (MALDI-TOF MS, Bruker, Germany).

### Antimicrobial susceptibility testing

Antimicrobial susceptibility testing was carried out according to the Kirby-Bauer disk diffusion technique using 16 antibiotics (Bio-Rad, France) (Supplementary Table S1). Reading of the results was done using the ADAGIO 93400 automated system (Bio-Rad, France) and interpreted as resistant (R), intermediate (I), and susceptible (S) based on the respective breakpoints for specific organisms in the European Committee on Antimicrobial Susceptibility Testing (EUCAST, 2021).

### Phenotypic detection of ESBLs

ESBL phenotype identification was carried out using MAST disks (Mast Group, UK) on all isolates (*n* = 648) that were non-susceptible to beta-lactam antibiotics such as cefotaxime, cefoxitin, cefepime, piperacillin/tazobactam, or meropenem in the Kirby-Bauer disk diffusion technique. The results were interpreted using the Mast Disks Combi D68C ESBL/AmpC calculator spreadsheet (Mast Group, UK) and reported as negative, positive, or inconclusive for ESBL or/and AmpC. Isolates with reports of “Further work required” or “Equivocal” or that grew toward all disks with below 9 mm of inhibition zone were grouped together as “inconclusive.”

### Detection of carbapenem resistance using Etest strips

All bacterial isolates that were intermediate or resistant to meropenem in the Kirby-Bauer disk diffusion method were tested with ertapenem Etest strips for *Enterobacterales* and meropenem Etest strips (both BioMérieux Deutschland GmbH) for non-lactose fermenting Gram-negative rods. According to EUCAST’s breakpoints for meropenem, an isolate was considered intermediate if the MIC value was between 2 and 8 mg/L and resistant when the MIC was greater than 8 mg/L. Bacterial isolates with MIC values greater than 0.5 mg/L were interpreted as resistant to ertapenem. Otherwise, all the remaining strains were considered susceptible to meropenem or ertapenem, respectively (EUCAST, 2021).

### Detection of carbapenemase encoding genes using PCR

The DNA was extracted from 3 to 5 fresh pure colonies of the respective bacterial isolate and extracted using High Pure PCR template preparation kit (Roche, Germany) following the manufacturer’s instruction. The quantity, purity, and concentration of the extracted DNA were measured by Nano-Drop ND-100 (Thermo Fisher Scientific, Wilmington, USA). Excluding the intrinsic carbapenem-resistant *S. maltophilia*, all the remaining isolates (*n* = 155) that were resistant to carbapenem antibiotics and/or showed inconclusive results in ESBL phenotypes by Mast disks (Mast Group, UK) were characterized by multiplex PCR to detect the carbapenemase encoding genes using specific primers and probes (Supplementary Table S2) used in previous studies (Kruttgen et al., 2011; Huang et al., 2012) and kindly provided by the molecular diagnostics of the Max von Pettenkofer Institute by Schubert S. and Gross B. Reference strains carrying *bla*OXA-48 (*K. pneumoniae* ATCC-BAA-2524), *bla*KPC (*E. coli* ATCC-1101362), and *bla*NDM (*K. pneumoniae* ATCC-BAA-2146) were used as positive controls.

### Statistical analysis

The data was entered and analyzed using Microsoft Office 2016 excel sheets and GraphPad Prism version 8.4.3. Tables and graphs were used to display the frequency of phenotypic antibiotic resistance patterns and the distribution of carbapenemase encoding genes among phenotypically carbapenem-resistant bacterial pathogens.

### Ethical considerations

The study was carried out with the approval of both Jimma University Institute of Health Institutional Review Board, Ethiopia (protocol numbers: IHRPGO/495/2018 & IHRPGO/1087/21) and the Ethics Committee of the Medical Faculty of Ludwig-Maximilians-Universität of Munich, Germany (Opinion No: 21–0157). Written informed consent was obtained from study participants and parents or guardians in case of neonates, infants, and children before enrollment in the study. All the information was kept confidential and recorded anonymously. The culture results were sent back timely to the treating physicians to provide the recommended medical attention to the respective patients.

## Results

### Frequency of Gram-negative bacterial isolates

A total of 1,794 clinical specimens were processed during the study period. Of these, 953 specimens collected from 894 patients were positive resulting in the isolation of 1,010 bacterial strains. The majority of isolates (846/1,010) were GNB, which were the only one included in the current study. A single bacterial pathogen was identified in 896 specimens, while two and three isolates were detected in the remaining 55 and 2 clinical samples, respectively. Overall, more than 30 different species of GNB were identified. The most commonly identified bacterial pathogen was *E. coli* accounting for 27% (231/846) of the GNB isolates, followed by *K. pneumoniae* 19% (163/846), *A. baumannii* complex 15% (126/846), and *E. cloacae* complex 13% (108/846) (Supplementary Table S3). More than 75% (643/846) of the GNB were isolated from admitted patients. Of these, 32% (206/643) were from the neonatal intensive care unit (NICU), 27% (184/643) from surgical, 27% (173/643) from pediatric, and 12% (80/643) from medical wards.

### Antimicrobial resistant pattern of Gram-negative bacteria

**Figure 1:**
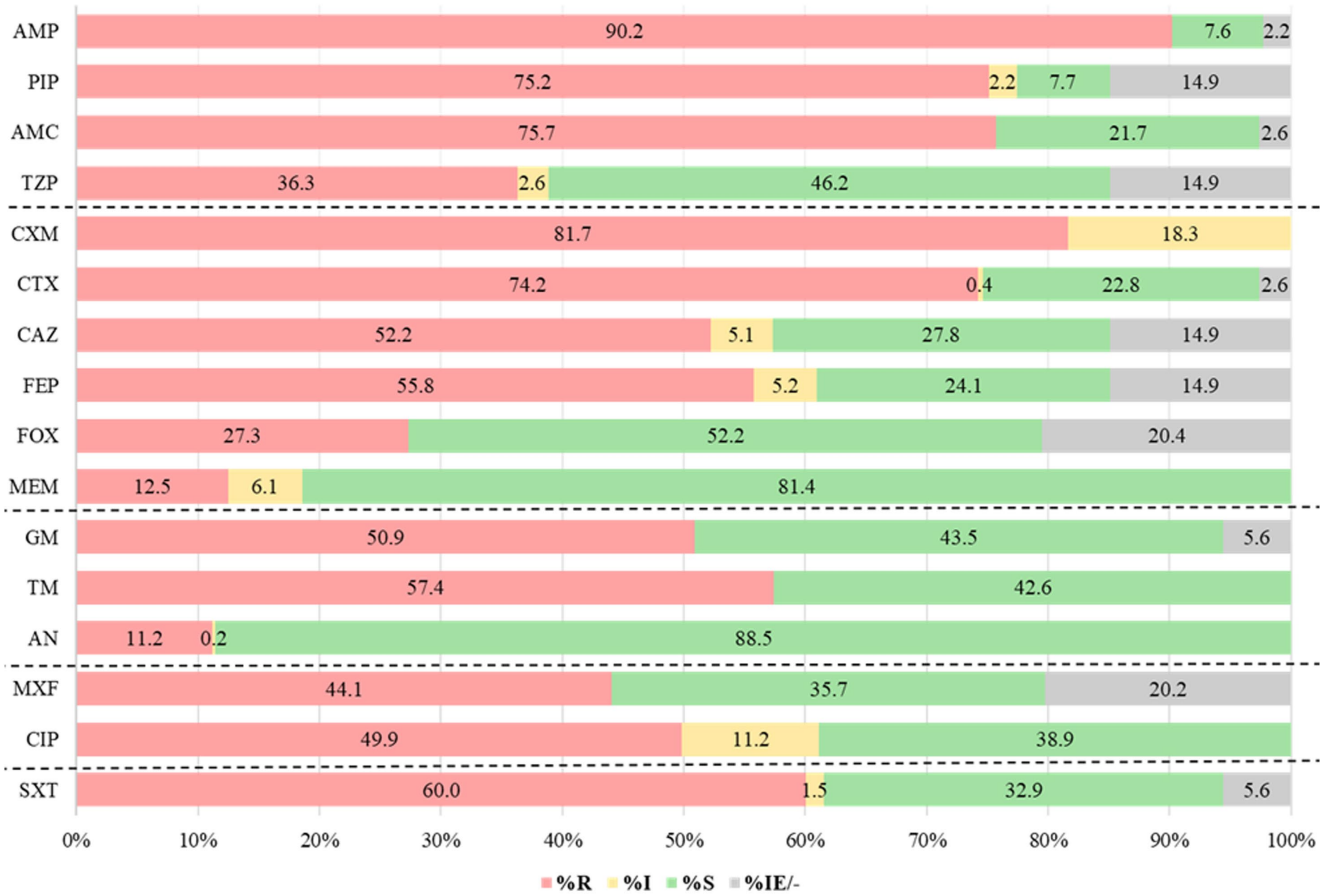
Antibiotic resistance patterns for Gram-negative bacteria (*n* = 846). AMP, ampicillin; PIP, piperacillin; AMC, amoxicillin-clavulanic acid; TZP, piperacillin-tazobactam; CXM, cefuroxime; CTX, cefotaxime; CAZ, ceftazidime; FEP, cefepime; FOX, cefoxitin; MEM, meropenem; GM, gentamicin; TM, tobramycin; AN, amikacin; MXF, moxifloxacin; CIP, ciprofloxacin; SXT, Trimethoprim- sulfamethoxazole; R, resistant; I, intermediate; S, susceptible, IE: insufficient evidence; and “–” No breakpoints.

In Kirby-Bauer disk diffusion technique, a remarkable prevalence of non-susceptibility was observed against cefuroxime, ampicillin, and piperacillin, with rates reaching 100% (846/846), 92% (763/827), and 91% (655/720) respectively. Among the tested antibiotics, meropenem and amikacin showed the least resistance, 18% (149/846) and 12% (97/846), respectively. The isolates also exhibited a high rate of resistance to trimethoprim-sulfamethoxazole (60%), aminoglycosides (11–57.4%), and fluoroquinolones (55.3–61.1%) (Figure 1).

### Prevalence of ESBL phenotypes

**Figure 2:**
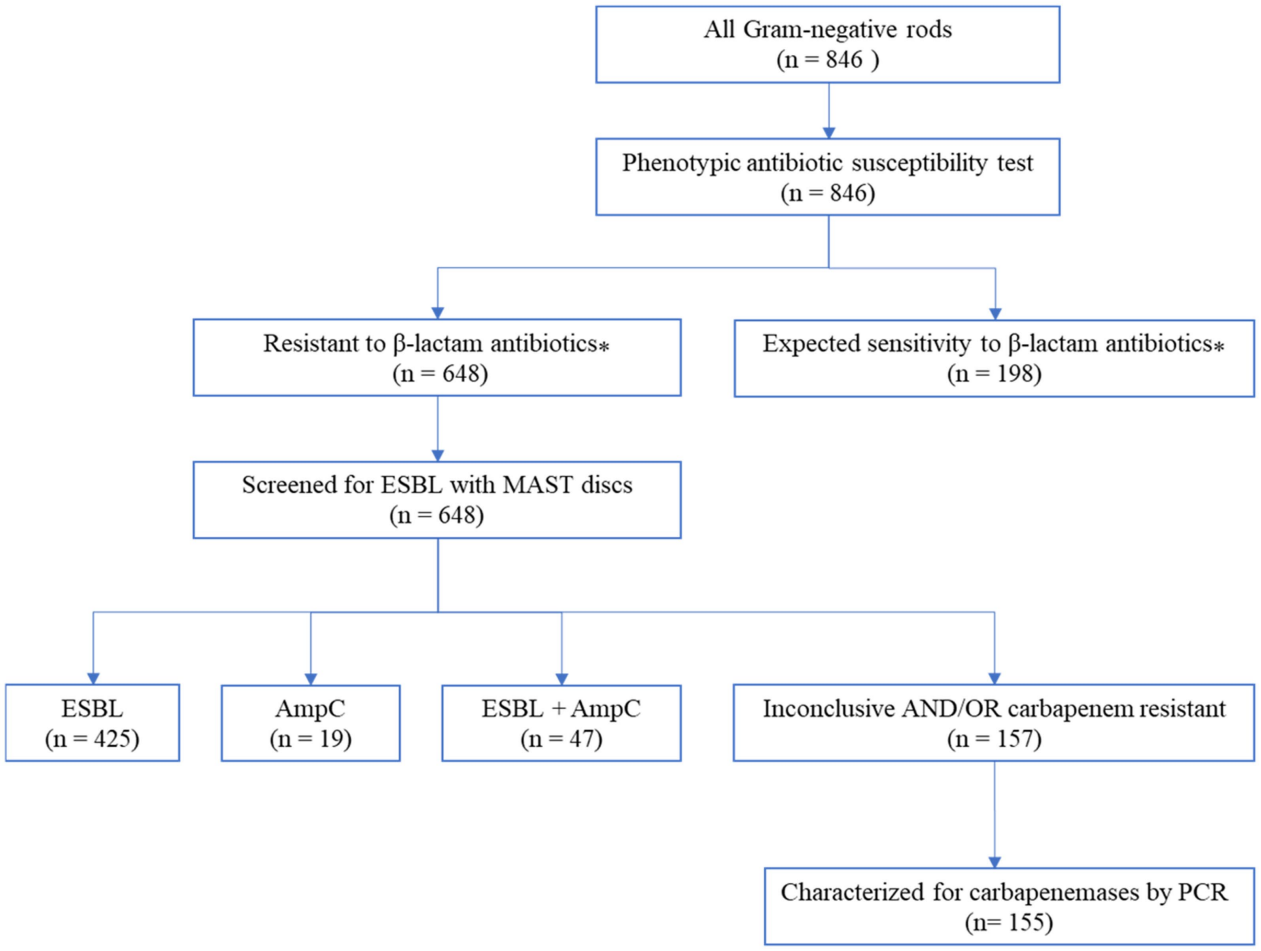
Flow diagram of the laboratory analysis to detect carbapenem-resistant Gram-negative bacteria. ^⁎^The antibiotic susceptibility test result to selected beta-lactam antibiotics such as cefotaxime, cefoxitin, cefepime, piperacillin/tazobactam, or meropenem; intrinsic resistances according to EUCAST are considered as expected; values with insufficient evidence according to EUCAST were not taken into account (EUCAST, 2021).

All 648 bacterial isolates that were non-susceptible (tested intermediate or resistant) to one of the β-lactam antibiotics were further analyzed for ESBL phenotypes using Mast disks (MAST group UK). The analysis revealed that 66% (425/648) of the isolates produced extended-spectrum beta-lactamases (ESBL), 7% (47/648) had both ESBL and AmpC phenotypes, and 3% (19/648) showed only an AmpC phenotype (Figure 2). The remaining 24% (157/648) of the isolates showed inconclusive results when read with Mast disks combi D68C ESBL/AmpC calculator spreadsheets (Mast group, UK).

More than 75% (491/648) of the isolates that showed resistance to β-lactam antibiotics in the disk diffusion technique were confirmed as ESBL and/or AmpC phenotypes by Mast disks (Mast group, UK). As shown in Table 1, all *Citrobacter* species*, K. oxytoca*, *Proteus* species, *S. marcescens, M. morganii, C. sakazakii*, *L. adecarboxylata*, *M. odoratimimus*, and *P. stuartii* were confirmed as ESBL producers. Furthermore, the prevalence of ESBL production was observed in 93% (127/137) of *K. pneumoniae*, 94% (134/142) of *E. coli*, and 97% (98/101) of *Enterobacter* isolates. The remaining 24% (157/648) of the isolates showed inconclusive results, primarily *A. baumannii* complex, and *P. aeruginosa* which accounted for 71% (87/122) and 98% (42/43) of the respective isolates as shown in Table 1.

**Table 1:** Proportion of ESBL phenotypes in Gram-negative bacteria (*n* = 648).

**Bacteria**	**ESBL**		**AMPC**		**ESBL and AMPC**		**Inconclusive**	
	***n***	**%**	***n***	**%**	***n***	**%**	***n***	**%**
*A. baumannii* complex (*n* = 122)	14	11.5	1	0.8	20	16.4	87	71.3
*Citrobacter* species (*n* = 8)	5	NA	3	NA	0	0.0	0	0.0
*Enterobacter* species (*n* = 101)	85	84.2	6	5.9	7	6.9	3	3.0
*E. coli* (*n* = 142)	119	83.8	6	4.2	9	6.3	8	5.6
*K. oxytoca* (*n* = 9)	9	NA	0	0.0	0	0.0	0	0.0
*K. pneumoniae* (*n* = 137)	120	87.6	2	1.5	5	3.6	10	7.3
*K. variicola* (*n* = 21)	13	61.9	0	0.0	5	23.8	3	14.3
*Proteus* species (*n* = 34)	34	100.0	0	0.0	0	0.0	0	0.0
*P. aeruginosa* (*n* = 43)	1	2.3	0	0.0	0	0.0	42	97.7
*Pseudomonas* species (*n* = 4)	2	NA	0	0.0	0	0.0	2	NA
*S. marcescens* (*n* = 14)	14	NA	0	0.0	0	0.0	0	0.0
*M. morganii* (*n* = 5)	5	NA	0	0.0	0	0.0	0	0.0
Other GNRs (*n* = 8)	4	NA	1	NA	1	NA	2*	NA
Total (*n* = 648)	425	65.6	19	2.9	47	7.3	157	24.2

Other Gram-negative rods (GNRs): *Cronobacter sakazakii* (1), *Leclercia adecarboxylata* (2), *Myroides odoratimimus* (2), *Providencia stuartii* (1), and *Stenotrophomonas maltophilia (2); NA, not applicable. Percentage is not calculated if the denominator is less than 20.

### Carbapenem minimum inhibitory concentrations

**Figure 3:**
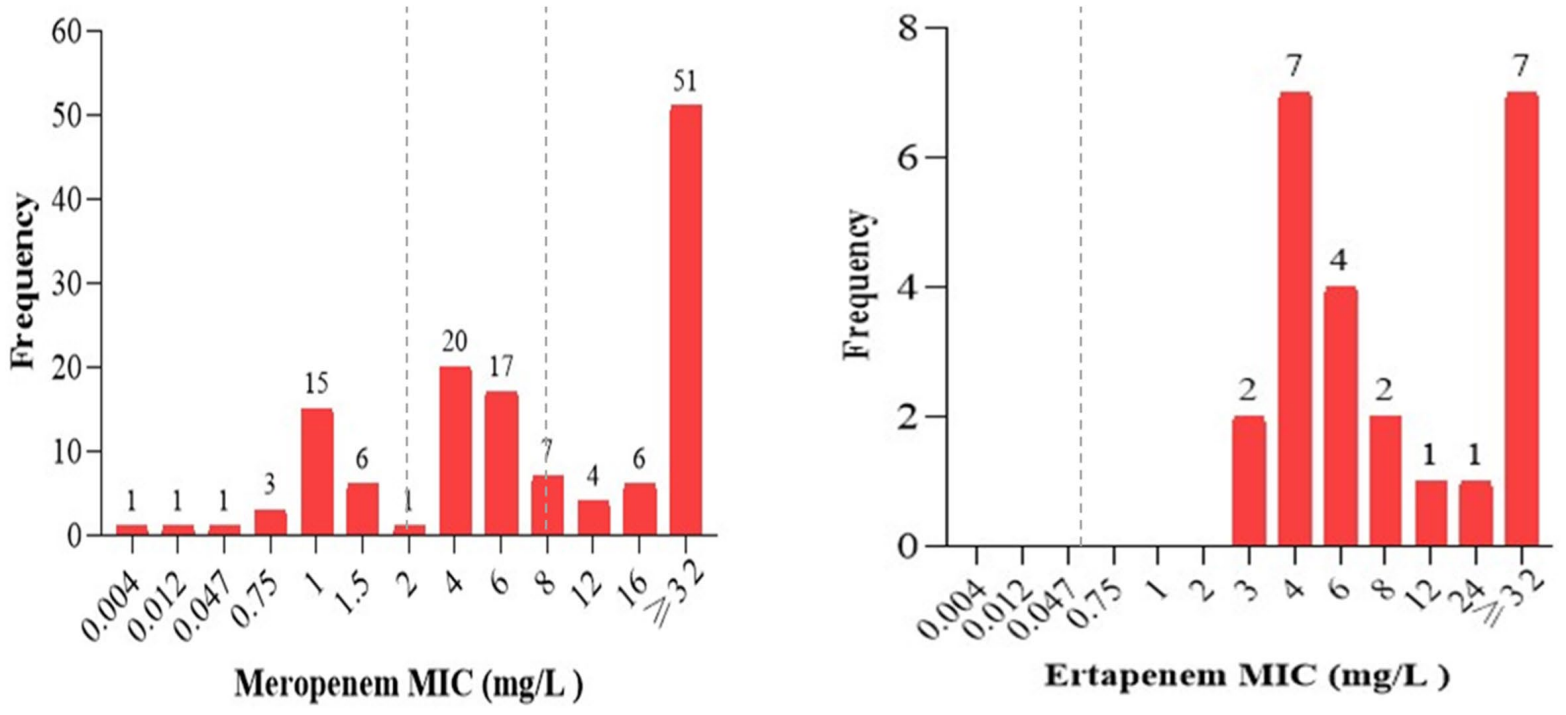
The frequency of carbapenem minimum inhibitory concentrations of all strains tested resistant in Kirby-Bauer disk diffusion or having inconclusive results in the Mast Disk assay. The MICs of meropenem ranging from (0–2 mg/L), (2–8 mg/L), and > 8 mg/L were interpreted as sensitive, intermediate, and resistant; ertapenem MIC values ≤0.5 mg/L and > 0.5 mg/L were interpreted as sensitive and resistant, respectively, as indicated in the broken lines according to EUCAST breakpoints (EUCAST, 2021).

The minimum inhibitory concentrations (MIC) of carbapenem antibiotics, specifically ertapenem for *Enterobacterales* and meropenem for non-lactose fermenting GNB, was determined using Etest strips. This was done for all isolates (*n* = 155) that were tested carbapenem-resistant in the Kirby-Bauer disk diffusion method and/or showed inconclusive results in the Mast disk analysis. Accordingly, 79% (105/133) of non-lactose fermenting isolates and 100% (24/24) of the lactose fermenting isolates showed intermediate or resistant phenotypes against meropenem or ertapenem Etest strip, respectively (Figure 3).

### Molecular epidemiology of carbapenemase-expression in Gram-negative bacteria

**Figure 4:**
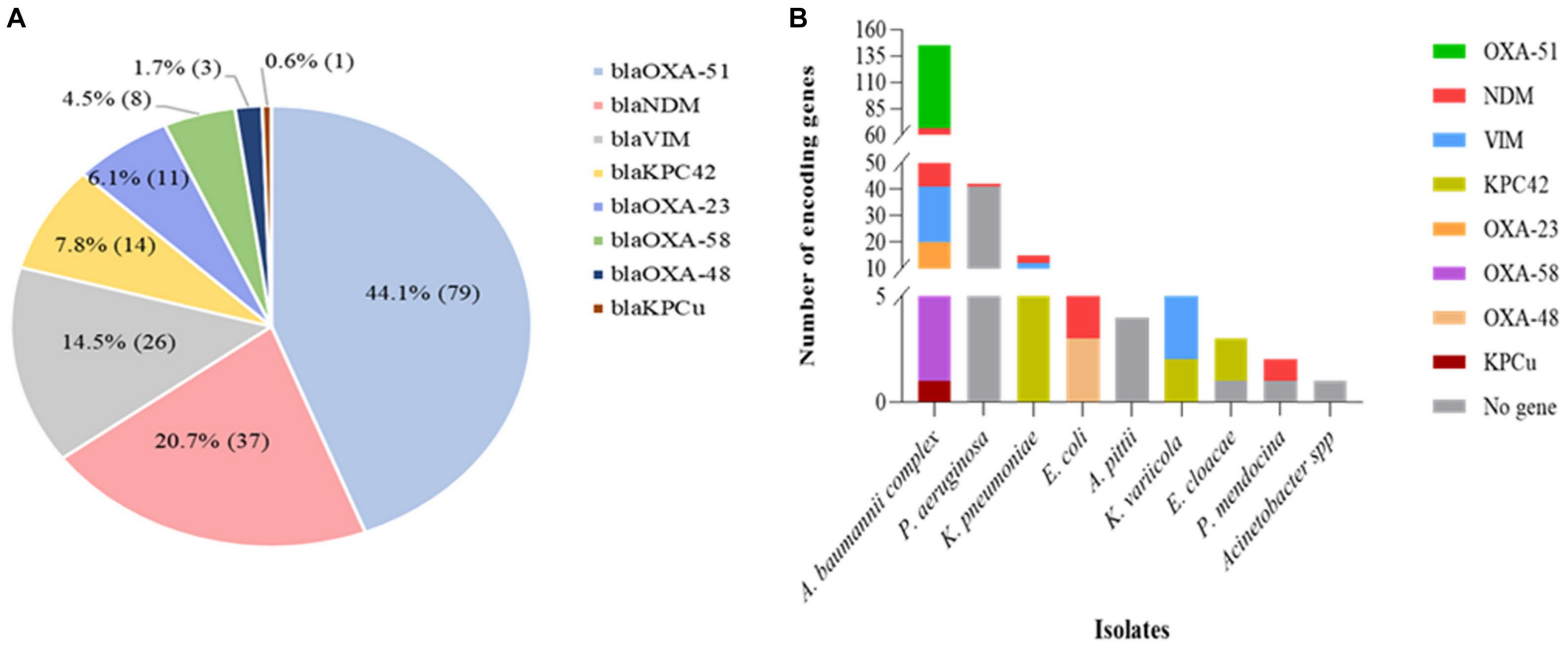
Distribution of carbapenemase encoding genes in various Gram-negative bacterial isolates with phenotypic resistance against carbapenems, as determined by PCR analysis. **(A)** The relative proportion of carbapenemase encoding genes (*n* = 179) as indicated in the pie chart. **(B)** The distribution of carbapenemase determinants in carbapenem-resistant isolates (*n* = 155). The PCR analysis revealed the presence of several types of carbapenemase determinants in many of the bacterial species. As a result, more than one carbapenemase determinant or mechanism of resistance was identified in 49 of the isolates.

The PCR analysis revealed that 69% (107/155) of the carbapenem non-susceptible isolates carried at least one carbapenemase-encoding gene, including both inherent and acquired genes. Among the acquired carbapenemase genes, the most frequently identified gene was *bla*NDM, constituting 21% (37/179) of the total detected genes. This was followed by *bla*VIM and *bla*KPC42, accounting for 15% (26/179), and 8% (14/179) respectively (Figure 4A). Regarding the distribution of carbapenemase-encoding genes, *bla*NDM was detected in various strains including *A. baumannii* (24), *E. coli* (6), *K. pneumoniae* (3), *K. variicola* (1), *P. aeruginosa* (1), *P. mendocina* (1) and *A. haemolyticus* (1). On the other hand, due to its intrinsic presence in *A. baumannii*, the *bla*OXA-51-like gene was exclusively found in *A. baumannii* strains (79) (Figure 4B). Conversely, no carbapenemase-encoding genes could be detected in 31% (48/155) of carbapenem-resistant isolates. *P. aeruginosa* was the most common, accounting for 85% (41/48) of them (Figure 4B).

Co-harboring of two or more acquired genes was observed in 31% (33/107) of the isolates, with *A. baumannii* being the predominant strain, accounting for 70% (23/33) of those isolates. Multiple gene coexistence was also detected in *A. haemolyticus* (1), *E. coli* (1), *K. pneumoniae* (5), and *K. variicola* (3) strains. The most common acquired coexisting genes were *bla*NDM + *bla*OXA-23, observed in 24% (8/33) of the isolates (Table 2).

**Table 2:** Frequency and distribution of carbapenemase-coding genes among Gram-negative bacteria (*n* = 107).

**Bacteria**	**AST using Etest strips**		**Carbapenem resistance genes (*n*)**	**Resistance strains** **% (*n*)**
	**Antibiotic**	**MIC (mg/L)**		
*Acinetobacter baumannii* complex (*n* = 81)	MP	≤2 (22)	OXA-51	38.3 (41)
		2–8 (19)		
		≤2 (4)	VIM + OXA-51	12.2 (13)
		2–8 (6)		
		>8 (3)		
		>8 (8)	NDM + OXA-51 + OXA-23	7.5 (8)
		2–8 (1)	NDM + OXA-51 + OXA-58	4.7 (5)
		>8 (4)		
		>8 (5)	NDM + OXA-51 + VIM	4.7 (5)
		2–8 (1)	NDM + OXA-51	2.8 (3)
		>8 (2)		
		>8 (1)	NDM + OXA-51 + OXA-58 + VIM	0.9 (1)
		>8 (1)	NDM + OXA-23	0.9 (1)
		>8 (1)	OXA-51 + OXA-23	0.9 (1)
		2–8 (1)	VIM + OXA-51 + OXA-58	1.9 (2)
		>8 (1)		
		>8 (1)	NDM + KPCu	0.9 (1)
*Acinetobacter haemolyticus* (*n* = 1)	MP	>8 (1)	NDM + OXA-23	0.9 (1)
*Enterobacter cloacae* (*n* = 2)	ETP	>0.5 (2)	KPC42	1.9 (2)
*E. coli* (*n* = 8)	ETP	>0.5 (5)	NDM	4.7 (5)
		>0.5 (2)	OXA-48	1.9 (2)
		>0.5 (1)	NDM + OXA-48	0.9 (1)
*Klebsiella pneumoniae* (*n* = 10)	ETP	>0.5(2)	KPC42	4.7 (5)
		>0.5(3)		
		>0.5(3)	KPC42 + NDM	2.8 (3)
		>0.5(2)	KPC42 + VIM	1.9 (2)
*Klebsiella variicola* (*n* = 3)	ETP	>0.5(2)	KPC42 + VIM	1.9 (2)
		>0.5 (1)	NDM + VIM	0.9 (1)
*Pseudomonas aeruginosa* (*n* = 1)	MP	>8 (1)	NDM	0.9 (1)
*Pseudomonas mendocina* (*n* = 1)	MP	>8 (1)	NDM	0.9 (1)

Interpretation: Meropenem, MIC value ≤ 2 mg/L → S, 2-8 mg/L → I, > 8 mg/L → R; and Ertapenem, MIC value ≤ 0.5 mg/L → S, >0.5 mg/L → R, and screening cut-off for both antibiotics MIC > 0.12 mg/L.

## Discussion

Our study revealed high proportions of ESBL and carbapenemase producing Gram-negative pathogens, primarily *E. coli*, *K. pneumoniae*, *E. cloacae* complex, *A. baumannii* complex, and *P. aeruginosa* in comparison to previous studies conducted worldwide (Chen et al., 2021; Jean et al., 2022). In most low-income countries, carbapenems are considered the last-resort antibiotics, as other antibiotics like colistin and polymyxin B are not available. Carbapenem-resistant infections are increasing at alarming rates worldwide (Hammoudi Halat and Ayoub Moubareck, 2020), and this trend is even worse in low-income countries (Stewardson et al., 2019) including Ethiopia (Sewunet et al., 2022; Tilahun et al., 2022). Inadequate infection prevention and control measures, lack of proper hand hygiene, insufficient isolation precautions, and limited regular AMR surveillance (Ali et al., 2018; Eshetu et al., 2019) contribute to this problem.

More than three-fourths (76.6%, 648) of the isolates were tested resistant to one or more beta-lactam antibiotics such as cefotaxime, cefoxitin, cefepime, piperacillin/tazobactam, or meropenem. Among all isolates, 59% (499/846) showed ESBL phenotypes, and 19% (157/846) were carbapenem-resistant phenotypically. Our findings indicate an increase in ESBL phenotypes in Jimma compared to previous reports of 50–51% in 2016 (Gashaw et al., 2018; Zeynudin et al., 2018). The observed high prevalence of ESBL-producing isolates could be explained by the high rate of nosocomial infections among hospitalized patients (Ali et al., 2018). The lack of proper infection prevention and control practices (Sastry et al., 2017; Maki and Zervos, 2021), along with horizontal gene transfer (Da Silva and Domingues, 2016) and the spread of resistant genes within local microbial populations may contribute to the high rate of beta-lactam resistance. Additionally, the high rates of *Acinetobacter* and *Pseudomonas* species which are intrinsically resistant to many beta-lactam antibiotics could explain this increase.

In previous studies conducted in Ethiopia, the rate of carbapenem resistance among Gram-negative rods was low ranging 1.7–15.1% (Misha et al., 2021; Tekele et al., 2021; Seman et al., 2022; Tilahun et al., 2022; Alemayehu et al., 2023). However, our findings showed an increase in resistance to carbapenems (18.6%). Our current study revealed high rates of phenotypic carbapenem resistance among *Acinetobacter* (71.3%) and *Pseudomonas* species (97.7%, 42/43), compared to a previous study conducted in the same area in 2016, where resistance rates were 56.4 and 7.3% for *Acinetobacter* and *Pseudomonas* isolates, respectively (Sewunet et al., 2022). This increase in resistance may be attributed to the increasing use of carbapenems at the hospital and poor infection control measures. Infections caused by such resistant isolates greatly limit the treatment options. Therefore, addressing the rising threat of carbapenemase-producing *Acinetobacter* and *Pseudomonas* species requires a multifaceted approach including the implementation of effective infection prevention and control measures, promotion of antimicrobial stewardship programs to ensure appropriate antibiotics use, and development of new antibiotics effective against these resistant strains (Mulani et al., 2019; Jean et al., 2022).

Additionally, it is important to identify the determinants of carbapenem resistance in bacterial pathogens. While many isolates express a carbapenemase, others may develop resistance due to other mechanisms such as porin loss (Atrissi et al., 2021). In our study, we investigated both the phenotypic resistance and the presence of carbapenemase genes. In *A. baumannii*, we found the presence of intrinsically encoded *bla*OXA-51-like genes, as well as the acquired *bla*NDM and *bla*KPC encoding genes. We did not investigate any regulatory phenotypes involved in increased expression of *bla*OXA-51-like enzymes, so we can only speculate on their role in the phenotypically resistant isolates, possibly in combination with permeability issues or efflux pumps. Nevertheless, in the case of *P. aeruginosa,* the observed carbapenem resistance could not be linked to the carbapenemases tested in the study. Instead, it is more likely that the resistance is due to porin loss as suggested by a previous study (Atrissi et al., 2021).

Similar to previous studies conducted in Egypt (Abouelfetouh et al., 2019) and South Africa (Anane et al., 2020), PCR analysis revealed that all *A. baumannii* isolates carried the *bla*OXA-51-like genes. In 13.6% (11/82) and 9.9% (8/82) of *Acinetobacter* strains, *bla*OXA-23-like and *bla*OXA-58-like genes were detected, respectively. The prevalence of *bla*OXA-51-like gene in our study was higher than reported in a previous study in Jimma (63.1%) (Sewunet et al., 2022). This can be explained by the higher proportion of *A. baumannii* strains that currently dominate nosocomial infections as compared to previous studies. All 79 *A. baumannii* isolates carried the intrinsic *bla*OXA-51-like gene, but 22 of them were phenotypically susceptible to meropenem according to the MIC values. This can be explained by the intrinsic low efficiency of *bla*OXA-51, which is not easily detected by phenotypic methods, as reported in previous studies (Hu et al., 2007; Nigro and Hall, 2018).

The New Delhi metallo-beta-lactamase (NDM), classified as group B in the Ambler classification, is an enzyme that can break down a wide range of beta-lactam antibiotics, including carbapenems. It was first reported in Ethiopia in 2017 in *A. baumannii* strains (Pritsch et al., 2017). Back then, it could only be detected in some isolates of *Acinetobacter baumannii*, with no evidence of its presence in other isolates. However, NDM is no longer limited to *Acinetobacter* species and has been found in various GNB, such as *K. pneumoniae*, *K. variicola*, *E. coli*, *P. aeruginosa*, and *P. mendocina* (Legese et al., 2022; Seman et al., 2022; Sewunet et al., 2022; Tufa et al., 2022). This enzyme is particularly concerning because it can rapidly spread between different bacterial species through horizontal gene transfer, leading to the emergence of extensively drug-resistant infections (Da Silva and Domingues, 2016). It is also frequently associated with other antibiotic resistance determinants and may be transferred alongside them. Our study detected the *bla*NDM gene in 34.6% of carbapenemase positive isolates, which is comparable to a study conducted in Kenya where 30% of the isolates carried the NDM gene (Villinger et al., 2022). The other commonly acquired carbapenemase gene identified in our study was *bla*KPC42, which was found in all carbapenem-resistant *K. pneumoniae* (10) and two of the three carbapenem resistant *K. variicola* strains. It has not been previously reported in Ethiopia but has been frequently reported in other parts of the world (Miranda et al., 2018).

Most of the *A. baumannii* isolates in our study harbored two (19) or three (21) carbapenemase genes. Moreover, five *K. pneumoniae* and three *K. variicola* isolates carried two carbapenemase genes. In total, 50 of the isolates carried multiple carbapenemase genes (*bla*OXA-51, *bla*NDM, *bla*VIM, *bla*OXA-23, *bla*OXA-58, *bla*KPC42, *bla*OXA-48, and *bla*KPCu), which is consistent with other studies conducted in Ethiopia where multiple carbapenemase determinants have been reported (Legese et al., 2022; Sewunet et al., 2022). In general, the prevalence of NDM in *Acinetobacter* and other GNB has been increasing globally in recent years (Sands et al., 2021; Awoke et al., 2022; Seman et al., 2022).

There are certain limitations to our study that should be considered when interpreting the results. First, the study was conducted in a single tertiary level facility, which may not fully represent the diversity of antimicrobial resistance patterns in the broader community or other healthcare settings in the region. Second, the PCR analysis was performed on isolates that were phenotypically resistant to carbapenems in the disk diffusion method and/or showed inconclusive results in the Mast disk analysis. This approach may have excluded some isolates with reduced carbapenem susceptibility that were not detected by the phenotypic resistance, potentially underestimating the true burden of carbapenem resistance in the study area. Third, we did not investigate if the resistance against carbapenems observed in some *A. baumannii* strains was due to overexpression of OXA-51 or other metabolic or regulatory changes such as loss of permeability or increased efflux.

## Conclusion

Our study demonstrated a high rate of carbapenem resistance among GNB, primarily in *Acinetobacter* species. The majority of this resistance was attributed to carbapenemases, probably along with other factors. Consequently, treating infections caused by these pathogens in this region may prove challenging due to limited treatment options. To address this issue, it is essential to revise treatment strategies in order to effectively manage infections caused by resistant strains. Moreover, it is imperative to uphold diligent surveillance, apply optimal infection prevention and control strategies, and promote antimicrobial stewardship practices to effectively manage and combat the dissemination of carbapenem-resistant bacteria.

## Data availability statement

The original contributions presented in the study are included in the article/Supplementary material, further inquiries can be directed to the corresponding author/s.

## Ethics statement

This study was approved by the Institutional Review Board (IRB) of Jimma University Institute of Health, Ethiopia and The Ethics Committee at the Medical Faculty of LMU Munich, Germany. Written informed consent was also obtained from patients, parents, or guardians prior to recruitment in the study.

## Author contributions

MG: Conceptualization, Data curation, Formal analysis, Investigation, Methodology, Project administration, Supervision, Writing – original draft, Writing – review & editing. EG: Conceptualization, Data curation, Funding acquisition, Supervision, Writing – review & editing. SA: Conceptualization, Supervision, Writing – review & editing. LG: Data curation, Writing – review & editing. TS: Writing – review & editing. BA: Data curation, Writing – review & editing. GF: Conceptualization, Funding acquisition, Supervision, Writing – review & editing. AK: Conceptualization, Funding acquisition, Supervision, Writing – review & editing. AW: Conceptualization, Data curation, Funding acquisition, Investigation, Methodology, Supervision, Writing – original draft, Writing – review & editing.

## References

[ref1] Abouelfetouh A. Torky A. S. Aboulmagd E. (2019). Phenotypic and genotypic characterization of Carbapenem-resistant *Acinetobacter baumannii* isolates from Egypt. Antimicro. Resist. Infect. Control 8, 1–9. doi: 10.1186/s13756-019-0611-6 PMC6868752 31832185

[ref2] Aleidan F. A. Alkhelaifi H. Alsenaid A. Alromaizan H. Alsalham F. Almutairi A.. (2021). Incidence and risk factors of carbapenem-resistant Enterobacteriaceae infection in intensive care units: a matched case–control study. Expert Rev. Anti-Infect. Ther. 19, 393–398. doi: 10.1080/14787210.2020.1822736 32930620

[ref3] Alemayehu E. Fiseha T. Gedefie A. Alemayehu Tesfaye N. Ebrahim H. Ebrahim E.. (2023). Prevalence of Carbapenemase-producing Enterobacteriaceae from human clinical samples in Ethiopia: a systematic review and Meta-analysis. BMC Infect. Dis. 23:277. doi: 10.1186/s12879-023-08237-5, PMID: 37138285 PMC10155349

[ref4] Ali S. Birhane M. Bekele S. Kibru G. Teshager L. Yilma Y.. (2018). Healthcare associated infection and its risk factors among patients admitted to a tertiary hospital in Ethiopia: longitudinal study. Antimicrob. Resist. Infect. Control 7, 1–9. doi: 10.1186/s13756-017-0298-5 29312659 PMC5755436

[ref5] Anane Y. A. Apalata T. Vasaikar S. Okuthe G. E. Songca S. (2020). Molecular detection of carbapenemase-encoding genes in multidrug-resistant *Acinetobacter baumannii* clinical isolates in South Africa. Int. J. Microb. 2020, 1–10. doi: 10.1155/2020/7380740 PMC7306865 32612659

[ref6] Atrissi J. Milan A. Bressan R. Lucafò M. Petix V. Busetti M.. (2021). Interplay of Opdp Porin and chromosomal carbapenemases in the determination of carbapenem resistance/susceptibility in *Pseudomonas aeruginosa*. Microbiol. Spectrum 9, E01186–E01121. doi: 10.1128/Spectrum.01186-21 PMC8557820 34585948

[ref7] Aurilio C. Sansone P. Barbarisi M. Pota V. Giaccari L. G. Coppolino F.. (2022). Mechanisms of action of Carbapenem resistance. Antibiotics 11:421. doi: 10.3390/antibiotics11030421, PMID: 35326884 PMC8944602

[ref8] Awoke T. Teka B. Aseffa A. Sebre S. Seman A. Yeshitela B.. (2022). Detection of Bla Kpc and Bla Ndm carbapenemase genes among *Klebsiella pneumoniae* isolates in Addis Ababa, Ethiopia: dominance of Bla Ndm. PLoS One 17:E0267657. doi: 10.1371/journal.pone.0267657, PMID: 35476721 PMC9045624

[ref9] Beshah D. Desta A. F. Woldemichael G. B. Belachew E. B. Derese S. G. Zelelie T. Z.. (2023). High burden of Esbl and carbapenemase-producing gram-negative Bacteria in bloodstream infection patients at a tertiary care hospital in Addis Ababa, Ethiopia. PLoS One 18:E0287453. doi: 10.1371/journal.pone.0287453, PMID: 37368908 PMC10298750

[ref10] Caston J. J. Cano A. Perez-Camacho I. Aguado J. M. Carratala J. Ramasco F.. (2022). Impact of ceftazidime/avibactam versus best available therapy on mortality from infections caused by Carbapenemase-producing Enterobacterales (Cavicor study). J. Antimicrob. Chemother. 77, 1452–1460. doi: 10.1093/jac/dkac049, PMID: 35187577

[ref11] Chen H. Jean S. Lee Y. Lu M. Ko W. Liu P. (2021). Carbapenem-resistant Enterobacterales in long-term care facilities: a global and narrative review. Front. Cell. Infect. Microbiol. 11:601968. doi: 10.3389/fcimb.2021.601968 33968793 PMC8102866

[ref12] Da Silva G. J. Domingues S. (2016). Insights on the horizontal gene transfer of Carbapenemase determinants in the opportunistic pathogen *Acinetobacter baumannii*. Microorganisms 4:29. doi: 10.3390/microorganisms4030029, PMID: 27681923 PMC5039589

[ref13] Das S. (2023). The crisis of carbapenemase-mediated carbapenem resistance across the human–animal–environmental Interface in India. Infect Diseases Now 53:104628. doi: 10.1016/j.idnow.2022.09.023, PMID: 36241158

[ref14] Di Carlo P. Serra N. Lo Sauro S. Carelli V. M. Giarratana M. Signorello J. C.. (2021). Epidemiology and pattern of resistance of gram-negative Bacteria isolated from blood samples in hospitalized patients: a single center retrospective analysis from southern Italy. Antibiotics 10:1402. doi: 10.3390/antibiotics10111402, PMID: 34827340 PMC8614669

[ref15] Dwomoh F. P. Kotey F. C. Dayie N. T. Osei M.-M. Amoa-Owusu F. Bannah V.. (2022). Phenotypic and genotypic detection of carbapenemase-producing Escherichia Coli and *Klebsiella pneumoniae* in Accra, Ghana. PLoS One 17:E0279715. doi: 10.1371/journal.pone.0279715, PMID: 36584159 PMC9803230

[ref16] Eshetu B. Gashaw M. Berhane M. Abdissa A. Mcclure E. M. Goldenberg R. L.. (2019). Intravenous fluid contaminated with *Klebsiella oxytoca* as a source of Sepsis in a preterm newborn: case report. Am. J. Infect. Control 47, 840–842. doi: 10.1016/j.ajic.2018.12.025, PMID: 30723029

[ref17] EUCAST (2021). European committee on antimicrobial susceptibility testing, breakpoint tables for interpretation of Mics and zone diameters. European Society Of Clinical Microbiology and Infectious Diseases: Basel

[ref18] Gashaw M. Berhane M. Bekele S. Kibru G. Teshager L. Yilma Y.. (2018). Emergence of high drug resistant bacterial isolates from patients with health care associated infections at Jimma University medical center: a cross sectional study. Antimicrob. Resist. Infect. Control 7, 1–8. doi: 10.1186/s13756-018-0431-0 30479751 PMC6245755

[ref19] Hammoudi Halat D. Ayoub Moubareck C. (2020). The current burden of carbapenemases: review of significant properties and dissemination among gram-negative Bacteria. Antibiotics 9:186. doi: 10.3390/antibiotics9040186, PMID: 32316342 PMC7235769

[ref20] Hu W. S. Yao S.-M. Fung C.-P. Hsieh Y.-P. Liu C.-P. Lin J.-F. (2007). An Oxa-66/Oxa-51-like carbapenemase and possibly an efflux pump are associated with resistance to imipenem in Acinetobacter baumannii. Antimicrob. Agents Chemother. 51, 3844–3852. doi: 10.1128/AAC.01512-06, PMID: 17724156 PMC2151406

[ref21] Huang X.-Z. Cash D. M. Chahine M. A. Nikolich M. P. Craft D. W. (2012). Development and validation of a multiplex Taqman real-time Pcr for rapid detection of genes encoding four types of class D carbapenemase in Acinetobacter baumannii. J. Med. Microbiol. 61, 1532–1537. doi: 10.1099/jmm.0.045823-0 22878252

[ref22] Jean S.-S. Harnod D. Hsueh P.-R. (2022). Global threat of Carbapenem-resistant gram-negative Bacteria. Front. Cell. Infect. Microbiol. 12:236. doi: 10.3389/fcimb.2022.823684 PMC8965008 35372099

[ref23] Kruttgen A. Razavi S. Imohl M. Ritter K. (2011). Real-time Pcr assay and a synthetic positive control for the rapid and sensitive detection of the emerging resistance Gene new Delhi Metallo-Β-Lactamase-1 (Bla Ndm-1). Med. Microbiol. Immunol. 200, 137–141. doi: 10.1007/s00430-011-0189-y 21350860

[ref24] Legese M. H. Asrat D. Mihret A. Hasan B. Mekasha A. Aseffa A.. (2022). Genomic epidemiology of Carbapenemase-producing and Colistin-resistant Enterobacteriaceae among Sepsis patients in Ethiopia: a whole-genome analysis. Antimicrob. Agents Chemother. 66, E00534–E00522. doi: 10.1128/aac.00534-22 35876577 PMC9380574

[ref25] Maki G. Zervos M. (2021). Health care-acquired infections in low- and middle-income countries and the role of infection prevention and control. Infect. Dis. Clin. 35, 827–839. doi: 10.1016/j.idc.2021.04.014, PMID: 34362546 PMC8331241

[ref26] Miranda I. F. Dos Santos M. L. Oliveira W. C. S. Oliveira M. C. (2018). *Klebsiella pneumoniae* Produtora De Carbapenemase Do Tipo Kpc: Disseminação Mundial E Situação Atual No Brasil. Brazil. J. Surg. Clin. Res. 25, 113–119.

[ref27] Misha G. Chelkeba L. Melaku T. (2021). Bacterial profile and antimicrobial susceptibility patterns of isolates among patients diagnosed with surgical site infection at a tertiary teaching hospital in Ethiopia: a prospective cohort study. Ann. Clin. Microbiol. Antimicrob. 20, 1–10. doi: 10.1186/s12941-021-00440-z 33971896 PMC8112062

[ref28] Mulani M. S. Kamble E. E. Kumkar S. N. Tawre M. S. Pardesi K. R. (2019). Emerging strategies to combat Eskape pathogens in the era of antimicrobial resistance: a review. Front. Microbiol. 10:539. doi: 10.3389/fmicb.2019.00539, PMID: 30988669 PMC6452778

[ref29] Nigro S. J. Hall R. M. (2018). Does the intrinsic Oxaab (Bla Oxa-51-like) gene of *Acinetobacter baumannii* confer resistance to Carbapenems when activated by Isaba1? J. Antimicrob. Chemother. 73, 3518–3520. doi: 10.1093/jac/dky334, PMID: 30124881

[ref30] Nordmann P. Poirel L. (2019). Epidemiology and diagnostics of Carbapenem resistance in gram-negative Bacteria. Clin. Infect. Dis. 69, S521–S528. doi: 10.1093/cid/ciz824, PMID: 31724045 PMC6853758

[ref31] Pritsch M. Zeynudin A. Messerer M. Baumer S. Liegl G. Schubert S.. (2017). First report on Bla Ndm-1-producing *Acinetobacter baumannii* in three clinical isolates from Ethiopia. BMC Infect. Dis. 17, 1–7. doi: 10.1186/s12879-017-2289-9 28249575 PMC5333390

[ref32] Rabaan A. A. Eljaaly K. Alhumaid S. Albayat H. Al-Adsani W. Sabour A. A.. (2022). An overview on phenotypic and genotypic characterisation of carbapenem-resistant enterobacterales. Medicina 58:1675. doi: 10.3390/medicina58111675, PMID: 36422214 PMC9696003

[ref33] Sands K. Carvalho M. J. Portal E. Thomson K. Dyer C. Akpulu C.. (2021). Characterization of antimicrobial-resistant gram-negative Bacteria that cause neonatal Sepsis in seven low-and middle-income countries. Nat. Microbiol. 6, 512–523. doi: 10.1038/s41564-021-00870-7, PMID: 33782558 PMC8007471

[ref34] Sastry S. Masroor N. Bearman G. Hajjeh R. Holmes A. Memish Z.. (2017). The 17th international congress on infectious diseases workshop on developing infection prevention and control resources for low-and middle-income countries. Int. J. Infect. Dis. 57, 138–143. doi: 10.1016/j.ijid.2017.01.040, PMID: 28216179 PMC7110576

[ref35] Seman A. Mihret A. Sebre S. Awoke T. Yeshitela B. Yitayew B.. (2022). Prevalence and molecular characterization of extended Spectrum Β-lactamase and carbapenemase-producing Enterobacteriaceae isolates from bloodstream infection suspected patients in Addis Ababa, Ethiopia. Infect. Drug Resist. 15, 1367–1382. doi: 10.2147/IDR.S349566, PMID: 35378892 PMC8976516

[ref36] Sewunet T. Asrat D. Woldeamanuel Y. Aseffa A. Giske C. G. (2022). Molecular epidemiology and antimicrobial susceptibility of *Pseudomonas* Spp. and *Acinetobacter* Spp. from clinical samples at Jimma Medical Center, Ethiopia. Front. Microbiol. 13:951857. doi: 10.3389/fmicb.2022.951857, PMID: 36204631 PMC9530197

[ref37] Shanmugakani R. K. Srinivasan B. Glesby M. J. Westblade L. F. Cárdenas W. B. Raj T.. (2020). Current state of the art in rapid diagnostics for antimicrobial resistance. Lab Chip 20, 2607–2625. doi: 10.1039/D0LC00034E, PMID: 32644060 PMC7428068

[ref38] Sikora A. Zahra F. (2020). Nosocomial infections. StatPearl. Available at: https://www.ncbi.nlm.nih.gov/books/NBK559312 32644738

[ref39] Stewardson A. J. Marimuthu K. Sengupta S. Allignol A. El-Bouseary M. Carvalho M. J.. (2019). Effect of Carbapenem resistance on outcomes of bloodstream infection caused by Enterobacteriaceae in low-income and middle-income countries (panorama): a multinational prospective cohort study. Lancet Infect. Dis. 19, 601–610. doi: 10.1016/S1473-3099(18)30792-8, PMID: 31047852

[ref40] Tekele S. G. Teklu D. S. Legese M. H. Weldehana D. G. Belete M. A. Tullu K. D.. (2021). Multidrug-resistant and Carbapenemase-producing Enterobacteriaceae in Addis Ababa, Ethiopia. Biomed. Res. Int. 2021, 1–10. doi: 10.1155/2021/9999638 34195291 PMC8214486

[ref41] Tenover F. C. Nicolau D. P. Gill C. M. (2022). Carbapenemase-producing *Pseudomonas aeruginosa*–an emerging challenge. Emerg. Microbes Infect. 11, 811–814. doi: 10.1080/22221751.2022.2048972, PMID: 35240944 PMC8920394

[ref42] Tilahun M. Gedefie A. Bisetegn H. Debash H. (2022). Emergence of high prevalence of extended-spectrum beta-lactamase and carbapenemase producing acinetobacter species and *Pseudomonas aeruginosa* among hospitalized patients at Dessie comprehensive specialized hospital, north-East Ethiopia. Infect. Drug Resist. 15, 895–911. doi: 10.2147/IDR.S358116, PMID: 35299856 PMC8921833

[ref43] Tufa T. B. Mackenzie C. R. Orth H. M. Wienemann T. Nordmann T. Abdissa S.. (2022). Prevalence and characterization of antimicrobial resistance among gram-negative Bacteria isolated from febrile hospitalized patients in Central Ethiopia. Antimicrob. Resist. Infect. Control 11:8. doi: 10.1186/s13756-022-01053-7, PMID: 35033191 PMC8761287

[ref44] Van Duin D. (2017). Carbapenem-resistant Enterobacteriaceae: what we know and what we need to know, vol. 8. Oxfordshire, UK: Taylor & Francis. 379–382. 10.1080/21505594.2017.1306621 PMC5477690 28402724

[ref45] Villinger D. Schultze T. G. Musyoki V. M. Inwani I. Aluvaala J. Okutoyi L.. (2022). Genomic transmission analysis of multidrug-resistant gram-negative Bacteria within a newborn unit of a Kenyan tertiary hospital: a four-month prospective colonization study. Front. Cell. Infect. Microbiol. 12:892126. doi: 10.3389/fcimb.2022.892126 36093198 PMC9452910

[ref46] Zeynudin A. Pritsch M. Schubert S. Messerer M. Liegl G. Hoelscher M.. (2018). Prevalence and antibiotic susceptibility pattern of Ctx-M type extended-Spectrum Β-lactamases among clinical isolates of gram-negative Bacilli in Jimma, Ethiopia. BMC Infect. Dis. 18, 1–10. doi: 10.1186/s12879-018-3436-7 30342476 PMC6196031

