# Supplementary material for: Molecular characterization of carbapenem-resistance in Gram-negative isolates obtained from clinical samples at Jimma Medical Center, Ethiopia
Source: Front Microbiol. 2024 Jan 24;15:1336387. doi: 10.3389/fmicb.2024.1336387 (PMC10848150; doi:10.3389/fmicb.2024.1336387)
Supplement: Supplementary file 1 [file Data_Sheet_1.docx]

# **Supplementary materials**

**Supplementary Table 1**: List of antibiotics tested by the Kirby-Bauer disc diffusion technique to all Gram-negative bacteria.

| **Sr. No** | **Antibiotics** | **Antibiotics code** | **Load (in μg)** |
| --- | --- | --- | --- |
|  | Ampicillin | AMP | 10 |
|  | Amoxicillin-clavulanic acid | AMC | 10 |
|  | Ceftazidime | CAZ | 30 |
|  | Cefotaxime | CTX | 30 |
|  | Cefuroxime | CXM | 30 |
|  | Cefepime | FEP | 30 |
|  | Cefoxitin | FOX | 30 |
|  | Meropenem | MEM | 10 |
|  | Gentamicin | GM | 10 |
|  | Amikacin | AN | 30 |
|  | Tobramycin | TM | 10 |
|  | Trimethoprim- sulfamethoxazole | SXT | 1.25/23.75 |
|  | Piperacillin | PIP | 30 |
|  | Piperacillin-tazobactam | TZP | 30 |
|  | Ciprofloxacin | CIP | 5 |
|  | Moxifloxacin | MXF | 5 |

**Supplementary Table 2:** Primer Sequences Used for Detection Extended-Spectrum Beta-Lactamase and Carbapenemase Genes

| **Genes** | **Sequence of primers from 5’→3’** |
| --- | --- |
| *bla*-KPC | Probe: 5’-**FAM**-CGCCGTGACGGAAAGCTTACAAAAACT-**BHQ-1**-3’  Primer fwd: 5’-TGCGCGCGATACCTCAT-3’  Primer rev: 5’-CCAGTGCAGAGCCCAGTGT-3’ |
| *bla*-KPCu &42 | Probe: 5’-**FAM**-CAGTCGGAGACAAAACCGGAACCTGC-**BHQ-1**-3’  Primer fwd: 5’-GCAGCGGCAGCAGTTTGTTGATT-3’  Primer rev: 5’-GTAGACGGCCAACACAATAGGTGC-3’  Primer fwd: 5’-ATGGCCGCTGGCTGGCTTTT-3’  Primer rev: 5’-GAGCGCGAGTCTAGCCGCAG-3’ |
| *bla*-NDM | Probe: 5’-**FAM**-TGGCATAAGTCGCAATCCCCGC-**BHQ-1**-3’  Primer fwd: 5’-GGCAAGCTGGTTCGACAAC-3’  Primer rev: 5’-GCTGGCGGTGGTGACTC-3’ |
| *bla*- IMP | Probe: 5’- **Cy5.5-**AAAAATCGAGAAGCTTGAAG- **BHQ-2**-3’  Primer fwd: 5’-CAGGAGCGGCTTTRCCTGAT-3’  Primer rev: 5’-TCGAACGATGTATGAACAWAAACA-3’  and  Probe: 5’- **HEX** -TGGTTACCTGAAAAGAAA- **BHQ-1-**3’  Primer fwd: 5’-AGGRCACACTCAAGATAACGTAGTG-3’  Primer rev: 5’-TCCGGTTTAACAAAGCAACCA-3’ |
| *bla*- VIM | Probe: 5’-**Cy5.5**-TGTCCGTGATGGTGATGAGTTGCT-**BHQ-2**-3’  Primer fwd: 5’-CCCGTCCAATGGTCTCA-3’  Primer rev: 5’-CCCACGCTGTATCAATCAA-3’ |
| *bla*-OXA-48 | Probe: 5’-**HEX**-TTACCCGCATCTACC-**BHQ-1**-3’  Primer fwd: 5’-CGGGCGAACCAAGCATT-3’  Primer rev: 5’-GGCGATCAAGCTATTGGGAAT-3’ |
| *bla*-OXA-23-like | Probe: 5’**-FAM-**CCAGTCTATCAGGAACTTGCGCGA**-BHQ1-**3’  Primer fwd: 5’-GACACTAGGAGAAGCCATGAAG-3’  Primer rev: 5’-CAGCATTACCGAAACCAATACG-3’ |
| *bla*-OXA-24 like | Probe: 5**’HEX-**AGTAACACCCATTCCCCATCCACTTTT**-BHQ1-**3’  Primer fwd: 5’-GATGACCTTGCACATAACCG-3’  Primer rev: 5’-CAGTCAACCAACCTACCTGTG-3’ |
| *bla*-OXA-51 like | Probe: 5’**-TexasRed-**ACTTGGGTACCGATATCTGCATTGCC**-BHQ2-**3’  Primer fwd: 5’-TGTCTAAGGAAGTGAAGCGTG-3’  Primer rev: 5’-AACTGTGCCTCTTGCTGAG-3’ |
| *bla*-OXA-58 like | Probe: 5’**-Cy5-**TGGACCAATACGACGTGCCAATTCT**-BHQ2-**3’  Primer fwd: 5’-AAGATTTTACTTTGGGCGAAGC-3’  Primer rev: 5’-CAACTTCCGTGCCTATTTGC-3’ |

**Supplementary Table 3**: Frequency of Gram-negative bacterial isolates obtained from AMR surveillance.

| **Bacterial isolates** | **Number (n)** | **Percent (%)** |
| --- | --- | --- |
| *Escherichia coli* | 231 | 27.3 |
| *Klebsiella pneumoniae* | 163 | 19.3 |
| *Acinetobacter baumannii* complex | 126 | 14.9 |
| *Enterobacter cloacae* complex | 108 | 12.8 |
| *Proteus mirabilis* | 62 | 7.3 |
| *Pseudomonas aeruginosa* | 43 | 5.1 |
| *Klebsiella variicola* | 38 | 4.5 |
| *Serratia marcescens* | 15 | 1.8 |
| *Klebsiella oxytoca* | 12 | 1.4 |
| *Proteus hauseri* | 8 | 0.9 |
| *Citrobacter freundii* | 7 | 0.8 |
| *Proteus vulgaris* | 6 | 0.7 |
| *Leclercia adecarboxylata* | 5 | 0.6 |
| *Morganella morganii* | 5 | 0.6 |
| Others | 17 | 2.0 |
| **Total** | **846** | 100% |

***Acinetobacter baumannii* complex**: *A. baumannii* (98), *A. pittii* (8), *A. ursingii* (3), *A. baylyi* (2), *A. lwoffii* (2), *A. haemolyticus* (1), *A. nosocomialis* (1), *A. seifertii* (1), *A. towneri* (1), and other *Acinetobacter* species (9); ***Enterobacter cloacae* complex**: *E. cloacae* (97), *E. asburiae* (5)*, E. bugandensis* (3), *E. kobei* (2), and *E. xiangfangensis* (1); **Others**: *Raoultella ornithinolytica* (3), *Cronobacter sakazakii* (2), *Myroides odoratimimus* (2), *Pseudomonas putida* (2), *Stenotrophomonas maltophilia* (2), *Citrobacter koseri* (1), *Escherichia hermannii* (1), *Providencia rettgeri* (1), *Providencia stuartii* (1), *Pseudomonas mendocina* (1), and *Pseudomonas* specie (1).
